# Estimation of radiation exposure of children undergoing superselective intra-arterial chemotherapy for retinoblastoma treatment: assessment of local diagnostic reference levels as a function of age, sex, and interventional success

**DOI:** 10.1007/s00234-020-02540-7

**Published:** 2020-08-29

**Authors:** Marcel Opitz, Denise Bos, Cornelius Deuschl, Alexander Radbruch, Sebastian Zensen, Selma Sirin, Michael Forsting, Nikolaos Bechrakis, Eva Biewald, Norbert Bornfeld, Petra Ketteler, Beate Timmermann, Martin Stuschke, Maja Guberina, Axel Wetter, Sophia Göricke, Nika Guberina

**Affiliations:** 1 Department of Diagnostic and Interventional Radiology and Neuroradiology, University Hospital Essen, Hufelandstrasse 55, 45147 Essen, Germany; 2 Department of Ophthalmology, University Hospital Essen, Hufelandstrasse 55, 45147 Essen, Germany; 3 Department of Pediatric Hematology and Oncology, University Hospital Essen, Hufelandstrasse 55, 45147 Essen, Germany; 4 Department of Particle Therapy, University Hospital Essen, West German Cancer Center, German Cancer Consortium (DKTK), Hufelandstrasse 55, 45147 Essen, Germany; 5 Department of Radiotherapy, University Hospital Essen, West German Cancer Center, Hufelandstrasse 55, 45147 Essen, Germany

**Keywords:** Radiation exposure, Retinoblastoma, Intra-arterial chemotherapy, Interventional neuroradiology

## Abstract

**Purpose:**

This study aims to determine local diagnostic reference levels (LDRLs) of intra-arterial chemotherapy (IAC) procedures of pediatric patients with retinoblastoma (RB) to provide data for establishing diagnostic reference levels (DRLs) in pediatric interventional radiology (IR).

**Methods:**

In a retrospective study design, LDRLs and achievable dose (AD) were assessed for children undergoing superselective IAC for RB treatment. All procedures were performed at the flat-panel angiography systems (I) ArtisQ biplane (Siemens Healthineers) and (II) Allura Xper (Philips Healthcare). Patients were differentiated according to age (A1: 1–3 months; A2: 4–12 months; A3: 13–72 months; A4: 73 months–10 years; A5: > 10 years), sex, conducted or not-conducted chemotherapy.

**Results:**

248 neurointerventional procedures of 130 pediatric patients (median age 14.5 months, range 5–127 months) with RB (68 unilateral, 62 bilateral) could be included between January 2010 and March 2020. The following diagnostic reference values, AD, and mean values could be determined: (A2) DRL 3.9 Gy cm^2^, AD 2.9 Gy cm^2^, mean 3.5 Gy cm^2^; (A3) DRL 7.0 Gy cm^2^, AD 4.3 Gy cm^2^, mean 6.0 Gy cm^2^; (A4) DRL 14.5 Gy cm^2^, AD 10.7 Gy cm^2^, mean 10.8 Gy cm^2^; (A5) AD 8.8 Gy cm^2^, mean 8.8 Gy cm^2^. Kruskal-Wallis-test confirmed a significant dose difference between the examined age groups (A2–A5) (*p* < 0.001). There was no statistical difference considering sex (*p* = 0.076) and conducted or not-conducted chemotherapy (*p* = 0.627). A successful procedure was achieved in 207/248 cases.

**Conclusion:**

We report on radiation exposure during superselective IAC of a pediatric cohort at the German Retinoblastoma Referral Centre. Although an IAC formally represents a therapeutic procedure, our results confirm that radiation exposure lies within the exposure of a diagnostic interventional procedure. DRLs for superselective IAC are substantially lower compared with DRLs of more complex endovascular interventions.

## Introduction

The role of pediatric interventional radiology (IR) procedures has increased over the last decade. These procedures are less common in the pediatric population but may comprise high radiation doses. In general, in pediatric patients, the developing organs and tissues are more sensitive to the harmful effects of radiation and the longer life expectancy increases the risk for developing radiation-induced cancer compared with that in adults receiving the same dose [1]. Nevertheless, pediatric international diagnostic reference levels (DRLs) are lacking for IR, especially for cerebral angiography.

DRLs are a vital element for dose monitoring and are globally accepted in order to achieve dose optimization in the clinical routine. They represent the 75th percentile of a dose distribution of a specific radiological procedure and may indicate whether the radiation dose lies within the normal range of a dose distribution at radiological departments [2, 3]. The achievable dose (AD) represents the 50th percentile of a dose distribution and may serve as another parameter for dose optimization [3, 4]. Therefore, the International Commission on Radiological Protection (ICRP) and the European Guidelines on Diagnostic Reference Levels for Pediatric Imaging are proclaiming the necessity for DRLs for pediatric patients [1, 5]. It is expected in the pediatric radiology community that pediatric DRLs will increase dose awareness and in the long term optimize the modification of equipment, technique, and imaging parameters.

In the last decade, intra-arterial chemotherapy (IAC) as an IR procedure has been applied increasingly in the clinical management of retinoblastoma. IAC is used both as primary and secondary treatment of retinoblastoma and it is reported to provide tumor control even in advanced-stage disease that might have previously required enucleation [6, 7]. Various studies demonstrated the possible benefits of this treatment, especially less systematic side effects and a lower rate of bulbus loss and relapse [8–10]. Thus, superselective IAC is more and more used in children with retinoblastoma (RB) for an eye preserving approach. However, superselective IAC always involves exposure to X-rays. This issue weighs even more considering the fact that the RB gene mutation itself represents a genetic predisposition to malignancy [11, 12].

Until now, little published data exists to our knowledge discussing radiation exposure of superselective, intra-arterial melphalan therapy in children with RB [13, 14]. Hence, the purpose of this study was to evaluate radiation exposure for children undergoing traditional guidewire-directed, superselective chemotherapy for RB treatment as a function of age, sex, and interventional success and to establish local diagnostic reference levels (LDRLs) as a function of age.

## Materials and methods

### Patient cohort

This retrospective study was approved by the internal ethical committee of our institution (20-9187-BO). Due to the retrospective nature of this study, no informed consent was required. All patient data were anonymized. The internal database of the radiology department of the University Hospital Essen was searched with an in-house-developed software for all IACs on retinoblastoma that were performed in the period between January 2010 and March 2020.

The cohort included patients with heritable and non-heritable RB, sporadic and familial RB, and uni-, bi- and trilateral tumors with ICRB grade B-E. Trilateral RB is a syndrome consisting of unilateral or bilateral hereditary RB associated with an intracranial neuroblastic tumor [15].

For cranial examinations, age is recommended as the grouping parameter in the European Guidelines on Diagnostic Reference Levels for Paediatric Imaging [5]. Following this, each examination was classified to one of five age groups (A1: 1–3 months; A2: 4–12 months; A3: 13–72 months; A4: 73 months −10 years; A5: > 10 years) depending on the patient age at the time of the interventional procedure. The distribution into the individual age groups is illustrated in Table 1.

**Table 1:** Number of applied and not applied IAC studies by age group

Age group	IAC applied	IAC not applied		
		Technical limitation	Collaterals to meningeal arteries	Alternative blood supply of the retina
A1	n/a	n/a	n/a	n/a
A2	74	5	4	2
A3	127	11	9	10
A4	4	n/a	n/a	n/a
A5	2	n/a	n/a	n/a
Total	207	16	13	12

*IAC*, intra-arterial chemotherapy; *n/a*, not applicable

### Procedure

**Fig. 1:**
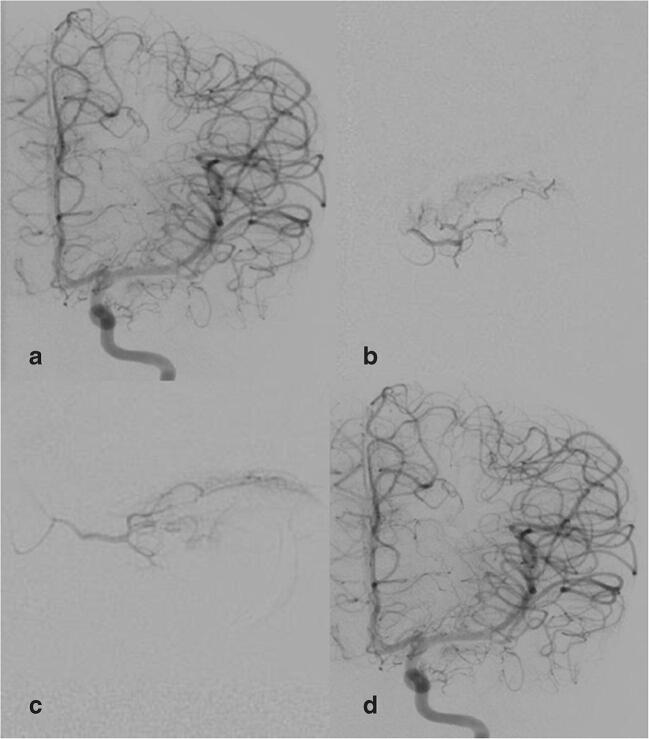
Serial angiogram of the internal carotid artery (ICA) in posterior/anterior and lateral projections is performed to visualize the cerebral and orbital angio-anatomy, here exemplary p.a. projection (**a**). This angiogram is used for smart mask for superselective catheterization of the ostium of the ophthalmic artery with a microcatheter. Injection of a contrast medium to confirm the correct position of the microcatheter and ascertain the lack of reflux into the internal carotid artery; p.a. (**b**) and lateral (**c**) projection. Injection of a weight-adapted chemotherapy under pressure control into the ophthalmic artery with a micro-perfusion pump over 30 min. After 15 min, a fluoroscopic control was conducted to confirm an unchanged microcatheter system positioning; at the end of the procedure prior to removal of the endovascular system, a final control angiogram of the ICA is performed in posterior/anterior and lateral projections in order to rule out vasospasm and intracerebral complication, here exemplary p.a. projection (**d**)

The standardized procedure is performed under general anaesthesia. A pediatric cardiologist provides transfemoral arterial access with a Doppler ultrasound–controlled needle in the Seldinger technique. The actual angiography examination by an interventional neuroradiologist comprises the following steps under fluoroscopic guidance (Fig. 1): (a) Placement of a 4-F guiding catheter into the cervical internal carotid artery and serial angiogram of the carotid artery in posterior/anterior and lateral projections is performed to visualize the cerebral and orbital angio-anatomy; (b) selective catheterization of the ostium of the ophthalmic artery with a microcatheter and injection of a contrast medium to confirm the correct position of the microcatheter and ascertain the lack of reflux into the internal carotid artery; hereby, choroid perfusion could be guaranteed, and meningeal collaterals excluded prior to intra-arterial melphalan therapy; (c) Injection of a weight-adapted chemotherapy under pressure control into the ophthalmic artery with a micro-perfusion pump over 30 min. Almost all our patients received a single-drug therapy with the cytostatic agent melphalan; only 2 patients received carboplatin alternatively. After 15 min, a fluoroscopic control was conducted to confirm an unchanged microcatheter system positioning; (d) at the end of the procedure prior to removal of the endovascular system, a final control angiogram is performed in posterior/anterior and lateral projections in order to rule out vasospasm and intracerebral complication. The standard procedure at our department is described in detail by Stenzel et al. [16]. The therapeutic regimen usually provides three sessions with an interval of 3–6 weeks and distance to previous polychemotherapy of at least 3 weeks [16].

### Biplanar angiography system

Since January 2010, all consecutive procedures performed at the (II) Allura Xper FD20/10 system (Philips Healthcare, Eindhoven, The Netherlands) and since November 2018 supplementary at the (I) Artis Q biplane angiography system (Siemens Healthineers, Erlangen, Germany) were included. Both X-ray units are equipped with automatic control dose rate systems. According to clinical standards, the frame rate in both systems was one image per second for a scan time of approximately 8 s.

The focus-to-skin distance (FSD) varied from 60 to 70 cm. The Artis Q biplane angiography system (I) has two 20-inch detectors, each with a maximum FOV (field of view) of 48 cm. The Allura Xper system (II) has one detector 20-inch with a maximum FOV of 48 cm and one 10-inch detector with a maximum FOV of 25 cm. Gonadal protection is routinely applied. To test system performance and stability over time, periodic quality controls were performed in both systems during maintenance visits.

### Endovascular system

Applied endovascular systems involved a Marathon-10 (Covidien/Medtronic, Inc., Mansfield, USA), HeadwayDuo (Microvention, Tustin, USA), or Echelon-10 microcatheter (Covidien/Medtronic, Inc., Mansfield, USA) as well as TransendEx-14 (Boston Scientific, Fremont, USA), Synchro-10 or Synchro-14 (both Stryker Neurovascular, Fremont, USA) microguidewires.

### Statistical analysis

The mean, median, 75th percentile, and standard deviation of DAP and median FT were calculated. The procedures were analyzed according to the five age groups described above. A *p* value lower than 0.05 was considered statistically significant. The descriptive statistics and statistical analysis were performed with the Statistical Package for Social Sciences v. 26.0 (SPSS Inc., New York, USA).

## Results

A total of 248 consecutive, neurointerventional procedures in 130 pediatric patients with retinoblastoma (68 unilateral; 62 bilateral; out of this 2 additional trilateral) were performed between January 2010 and March 2020. Of the 62 patients with bilateral RB, six patients received both left and right IAC in independent sessions. Patients received image-guided intra-arterial chemotherapy, either melphalan or carboplatin. The applied dose of melphalan (median 3 mg, 2.5–7.5 mg) as well as of carboplatin (30–40 mg) was adapted to weight and age. The median age of the patient cohort was 14.5 months (range, 5–127 months).

**Fig. 2:**
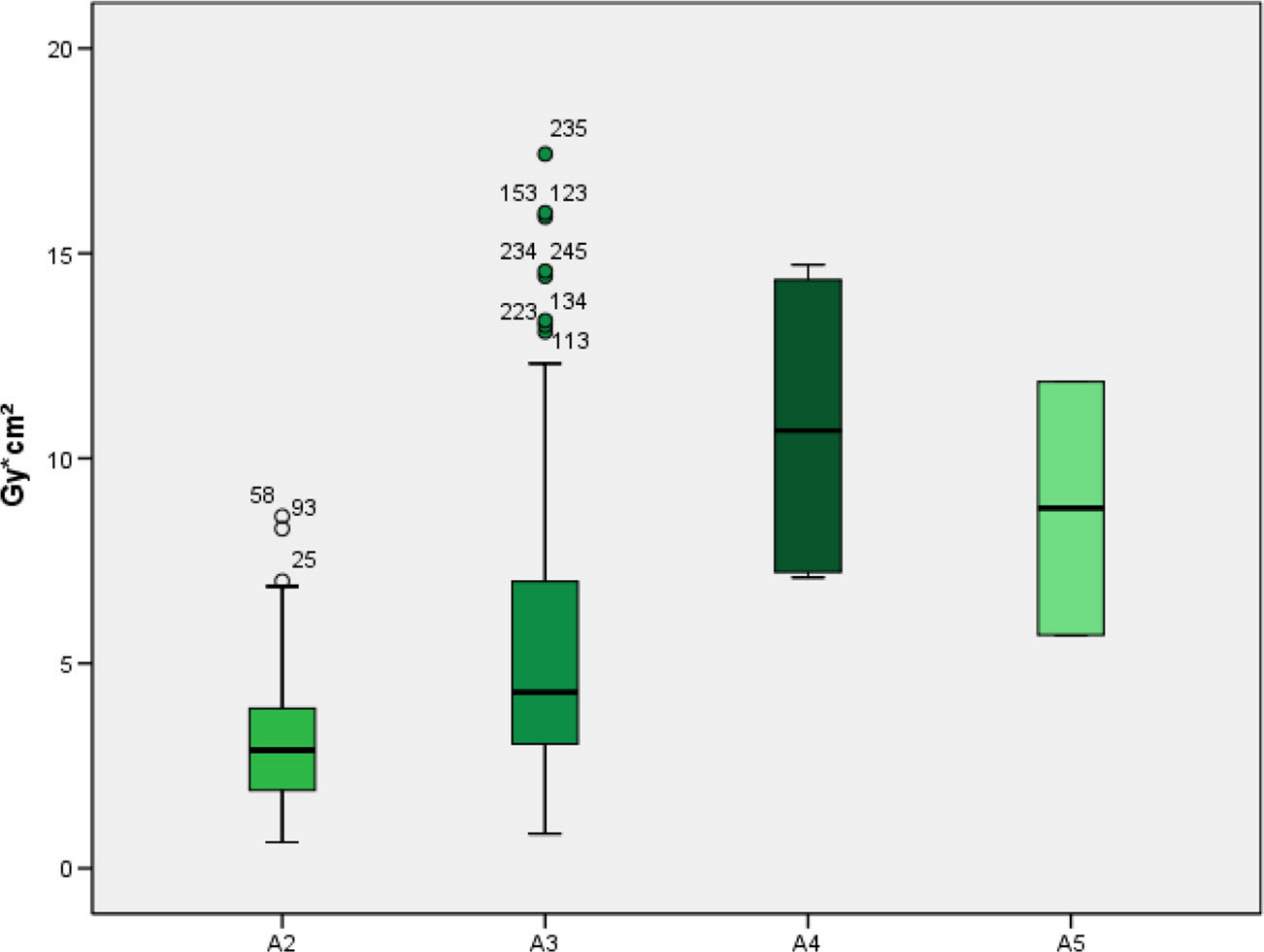
Radiation exposure differentiated according to age groups (A2–A5) performed at the flat-panel angiography system (I) Artis Q biplane (Siemens Healthineers, Erlangen, Germany) and (II) Allura Xper (Philips Healthcare, Eindhoven, The Netherlands)

**Fig. 3:**
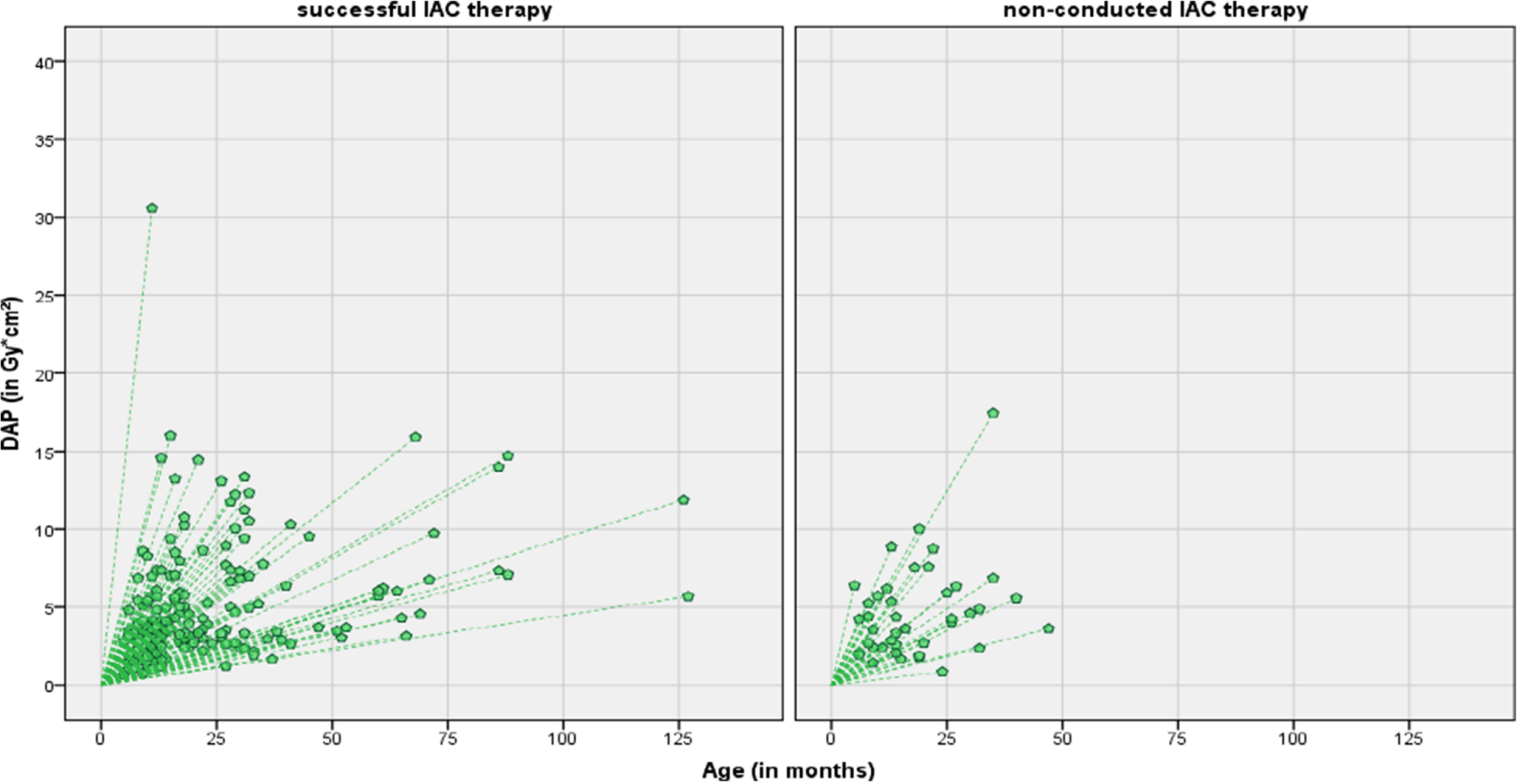
Scatter plot delineating dose area product (Gy cm²) of neurointerventional procedures as a function of age (in months) differentiated according to a successful (left) or non-conducted IAC therapy (right)

**Fig. 4:**
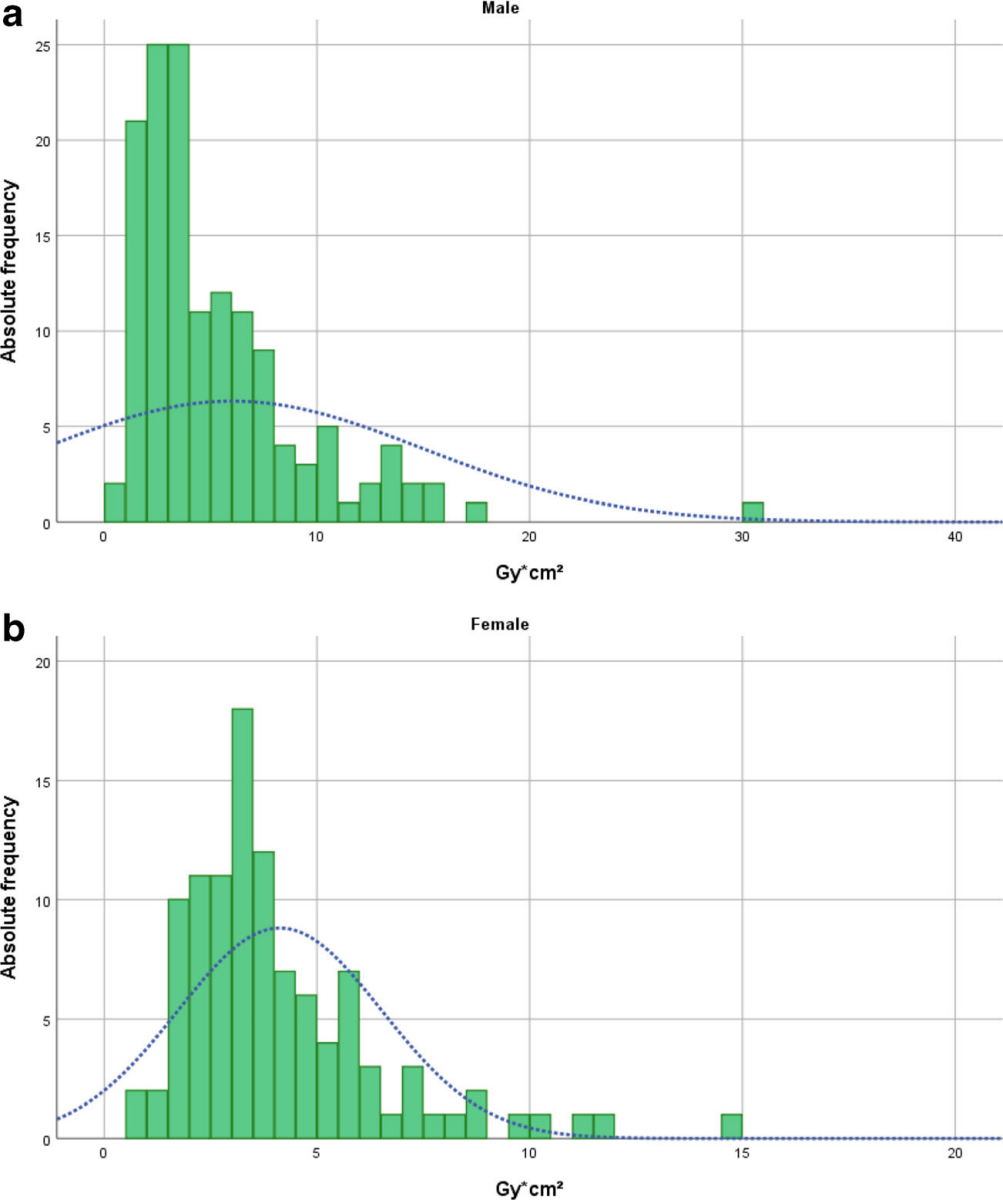
**a**, **b** Histogram distribution of dose area product (Gy cm²) for intra-arterial chemotherapy applications as a function of sex. **a** Upper histogram male patients with retinoblastoma and **b** lower histogram female patients (*p* = 0.076); the blue curve highlights distribution graph

Radiation exposure was distributed as follows: (A2) DRL 3.9 Gy cm^2^, AD 2.9 Gy cm^2^, mean 3.5 Gy cm^2^; (A3) DRL 7.0 Gy cm^2^, AD 4.3 Gy cm^2^, mean 6.0 Gy cm^2^; (A4) DRL 14.5 Gy cm^2^, AD 10.7 Gy cm^2^, mean 10.8 Gy cm^2^; (A5) AD 8.8 Gy cm^2^, mean 8.8 Gy cm^2^ (Table 2). Kruskal-Wallis-test confirmed a significant dose difference between the examined age groups (A2–A5) (*p* < 0.001) (Fig. 2). No statistical difference for conducted or not-conducted chemotherapy (*p* = 0.627) (Fig. 3) and sex (*p* = 0.076) (Fig. 4a, b) was found. The average number of individual superselective IAC administrations was 1.984 (median 1.00). A successful neurointerventional procedure was achieved in 207/248 (83.5%) cases. In 41/248 sessions (16.5%) the therapeutic angiography had to be interrupted without injecting IAC for the following reasons: (1) significant collaterals to meningeal arteries (13 patients), (2) technical failure of ophthalmic artery catheterization (16 patients), or (3) retina blood supply from collaterals different to the ophthalmic artery (12 patients). Only in one case was there a periprocedural complication after intra-arterial melphalan application in terms of thromboembolism.

**Table 2:** Number of IAC studies with median, mean, and 75th percentile of total DAP as a function of age group

Age group	Device		Total	Total DAP (Gy cm^2^)			T (min)
	I	II	*n*	Median	Mean	75th percentile	Median
A1	n/a	n/a	n/a	n/a	n/a	n/a	n/a
A2	9	76	85	2.9	3.5	3.9	7 min 41 s
A3	15	142	157	4.3	6.0	7.0	7 min 52 s
A4	n/a	4	4	10.7	10.8	14.5	6 min 20 s
A5	n/a	2	2	8.8	8.8	n/a	4 min 47 s

*DAP*, dose area product in gray per square centimeter; *T*, fluoroscopic time in minutes (median); *n*, number of studies; *n/a*, not applicable

## Discussion

Here, we present LDRLs of children undergoing superselective IAC for RB treatment at the German Retinoblastoma Referral Centre. Radiation exposure is of particular concern in children with heritable RB as they face a lifelong increased predisposition to second primary malignancies [17, 18].

The presented results show that the local diagnostic reference values and the achievable dose for superselective IAC therapy are substantially lower compared with DRLs of more complex endovascular interventions like thrombus aspiration (DRL 179.99 Gy cm^2^) or aneurysm coiling (DRL 249.99 Gy cm^2^) in adults, published by the Federal Office for Radiation Protection (Neuherberg, Germany) [19]. However, pediatric radiation dose is underestimated by the “one-size-fits-all” model [17]. Though several studies discussed a higher susceptibility of children to ionizing radiation [17, 18, 20–23], few authors addressed the issue of radiation exposure of pediatric interventional procedures at all, but in particular of superselective, intra-arterial melphalan therapies [13, 14] so far. The patient cohort described in these studies is limited to 11 [13] to 21 [14] sessions (including 8–16 patients). Vijayakrishnan et al. [13] described their preliminary data shortly after introducing this new technique [24]. Hence, it has to be postulated that they were able to reduce radiation exposure with increasing experience. Gobin et al. reported on only 14 superselective, intra-arterial melphalan therapies via the ophthalmic artery; 2 were performed via the middle meningeal artery and 5 in balloon technique [14]. Furthermore, radiation exposure was assessed by determining organ doses. This allows individual risk stratification. However, it is not practical in the clinical routine to compare radiation exposure of different devices and at different sites. Cooke et al. focused on the lens dose and the entrance skin dose in a small cohort (30 administrations) [25]. A recent study compared two techniques for ophthalmic chemotherapy administration [26]. The traditional guidewire-directed technique and the microcatheter-only technique as previously described by Gobin et al. [8], but with the addition of continuous verapamil infusion [26]. The authors focused on mean doses instead of on the DRLs.

The question about radiation exposure is of timeless concern in medical imaging [27], particularly in pediatric patients, who are generally more sensitive to radiation exposure [28]. Our analysis revealed that the DAP was not significantly higher during interventions in which an IAC could not be conducted due to an unsuccessful microcatheter and guide wire placement in the ophthalmic artery or meningeal anastomoses. However, all cases in which a chemotherapy could not be performed eventually result in an “unnecessary” radiation exposure. There was no difference of DAP concerning applied endovascular system.

In this context, DRLs are one of the main operational tools for detecting and optimizing image procedures in order to protect patients in radiological imaging. The DRLs were first recommended by the International Commission on Radiological Protection (ICRP) in 1991 [29] and some years later, in 1997, introduced in the European legislation by the Medical Exposure Directive 97/43/Euratom [30]. Since the Council Directive 2013/59/Euratom, all member states shall ensure that the established DRLs are regularly reviewed and used for optimization of protection [31]. Moreover, the Basic Safety Standards Directive expanded the application of DRLs to interventional radiology (IR) procedures [31]. Currently, only a few countries have set DRLs for pediatric examinations, and especially for pediatric interventional procedures, DRLs are rare. There are only few publications concerning patient dose for pediatric interventional cardiology [32–35]. For pediatric non-cardiologic interventional procedures, data is even scarcer. There is only one multicenter study from France publishing reference levels of three interventional neuroradiological procedures (cerebral digital subtraction angiography (DSA), embolization of brain arteriovenous malformation (bAVM), and percutaneous sclerotherapy of head and neck superficial vascular malformation (SVM)) [36]. Authors differentiated patient collective to different age groups: younger than 2 years (A1), aged 2–7 years (A5), 8–12 years (A10), and 13–18 years (A15). According to the study results, radiation exposures for a standard DSA were 4, 18, 12, and 32 Gy cm^2^, for bAVM 33, 70, 105, and 88 Gy cm^2^, and for SVM 350, 790, 490, and 248 m Gy cm^2^ in groups A1, A5, A10, and A15, respectively. Although an IAC formally represents a therapeutic procedure, our results confirm that radiation exposure lies within the exposure of a diagnostic interventional procedure.

Despite the fact that vein of Galen aneurysmal malformations represent the most common form of symptomatic cerebrovascular malformation in the early childhood, data on radiation exposure of therapeutic procedures in this special cohort are scarce. Curtis et al. examined the interventional procedure of a vein of Galen embolization of a 10-week-old by using a fluoroscopy fade technique [37]. The fluoroscopy fade technique is believed to comprise a lower contrast medium amount and a lower radiation exposure compared with the traditional road map–guided procedure. According to Curtis et al., the fluoroscopy fade technique in the vein of Galen embolization of the 10-week-old led to a skin entrance exposure of 480 mGy [37]. On the other hand, McParland reports a mean skin entrance dose of 100–110 mGy for standard p.a. and lateral projections as well as 340 mGy for standard embolization procedures [38]. Orbach et al. examined a total of 175 pediatric neurointerventions between September 2006 and July 2010 as well as 180 cases between July 2010 and June 2012 [39]. The examined neurointerventional procedures comprised several cerebrovascular pathologies such as brain AVM, pial fistulas, aneurysms, dural fistulas, and extracranial AVM or AVF, which were present in all age groups. The vein of Galen malformations were exclusively present in the < 1-year and 1- to 2-year age groups. According to Orbach et al., the maximal skin dose reached 372.9 mGy in the < 1-year-olds and 443.5 mGy in the1–2-year-olds [39].

For IR procedures, the patient dose depends on several factors, including the age and size of the patient, the complexity of the specific situation, and the experience of the medical staff. In general, therapeutic procedures have been reported to yield higher dose than diagnostic procedures [36]. Therefore, pediatric DRLs should be defined separately for specified diagnostic or therapeutic procedures [5].

## Conclusion

RB represents one of the most frequent ocular malignancies in childhood. This is the first data acquisition of radiation exposure during superselective intra-arterial melphalan therapy of a pediatric cohort at a Retinoblastoma Referral Centre. Even if an IAC formally represents a therapeutic procedure, our results confirm that radiation exposure lies within the exposure of a diagnostic interventional procedure. Diagnostic reference values and the achievable dose for superselective IAC therapy are substantially lower compared with DRLs of more complex endovascular interventions. The examination evaluation of radiation exposure in a larger population and the comparison to international standards are the next necessary steps for the determination of national diagnostic reference levels (NDRLs) and European diagnostic reference levels (EDRLs) and to obtain a sufficient and reliable basis for implementing international pediatric DRLs which may ultimately contribute to radiation dose optimization in this rare but real entity.

## Abbreviations

AD: Achievable dose
DRLs: Diagnostic reference levels
DAP: Dose area product
EDRLs: European diagnostic reference levels
FOV: Field of view
IR: Interventional radiology
LDRLs: Local diagnostic reference levels
NDRLs: National diagnostic reference levels
RB: Retinoblastoma
